# Outcomes of patients admitted to the ICU for acute stroke: a retrospective cohort

**DOI:** 10.1186/s12871-022-01777-4

**Published:** 2022-07-25

**Authors:** Thibaut Carval, Charlotte Garret, Benoît Guillon, Jean-Baptiste Lascarrou, Maëlle Martin, Jérémie Lemarié, Julien Dupeyrat, Amélie Seguin, Olivier Zambon, Jean Reignier, Emmanuel Canet

**Affiliations:** 1 Service de Médecine Intensive Réanimation, Centre Hospitalier Universitaire de Nantes, Centre Hospitalier Universitaire Hôtel-Dieu, 30 Bd. Jean Monnet, 44093 Nantes Cedex 1, Nantes, France; 2 Service de Neurologie, Centre Hospitalier Universitaire de Nantes, Université de Nantes, Nantes, France

**Keywords:** Stroke, Intensive care unit, Mechanical ventilation, Coma, Disability

## Abstract

**Background:**

Although acute stroke is a leading cause of morbidity and mortality worldwide, data on outcomes of stroke patients requiring ICU admission are limited. We aimed to identify factors associated with a good neurological outcome (defined as a modified Rankin Scale score [mRS] of 0–2) 6 months after ICU admission.

**Methods:**

We retrospectively studied consecutive patients who were admitted to the ICU of a French university-affiliated hospital between January 2014 and December 2018 and whose ICD-10 code indicated acute stroke. Patients with isolated subarachnoid hemorrhage or posttraumatic stroke were excluded.

**Results:**

The 323 identified patients had a median age of 67 [54.5–77] years; 173 (53.6%) were male. The main reasons for ICU admission were neurological failure (87%), hemodynamic instability (28.2%), acute respiratory failure (26%), and cardiac arrest (5.3%). At ICU admission, the Glasgow Coma Scale score was 6 [4–10] and the SAPSII was 54 [35–64]. The stroke was hemorrhagic in 248 (76.8%) patients and ischemic in 75 (23.2%). Mechanical ventilation was required in 257 patients (79.6%). Six months after ICU admission, 61 (19.5%) patients had a good neurological outcome (mRS, 0–2), 50 (16%) had significant disability (mRS, 3–5), and 202 (64.5%) had died; 10 were lost to follow-up. By multivariable analysis, factors independently associated with not having an mRS of 0–2 at 6 months were older age (odds ratio, 0.93/year; 95% confidence interval, 0.89–0.96; *P* < 0.01) and lower Glasgow Coma Scale score at ICU admission (odds ratio, 1.23/point; 95% confidence interval, 1.07–1.40; *P* < 0.01).

**Conclusions:**

Acute stroke requiring ICU admission carried a poor prognosis, with less than a fifth of patients having a good neurological outcome at 6 months. Age and depth of coma independently predicted the outcome.

## Background

Stroke is a common event and a major cause of hospitalization, disability, and mortality worldwide [1]. Major therapeutic advances have occurred in the past decade, including the development of dedicated stroke units, the introduction of reperfusion therapy and interventional neuroradiology, and the performance of acute neurosurgical interventions in selected patients [2–4]. In clinical trials, these treatments decreased mortality and improved disability-free survival [5–7].

However, a growing number of stroke patients require ICU admission for either neurological monitoring or the management of stroke complications, with 10–30% becoming critically ill [8–10]. In addition, stroke patients without treatment options are increasingly being admitted to the ICU to facilitate organ donation. Among patients with stroke, significant differences exist between those admitted to ICUs and those admitted to neurological wards or stroke units. The ICU group is characterized by greater neurological severity, as measured using validated tools (e.g., the National Institutes of Health Stroke Scale, NIHSS [11]); moderate-to-severe consciousness impairment; a need for mechanical ventilation in many cases [9, 12]; and high hospital mortality. Data are limited on the potential benefits of acute-phase stroke therapy in ICU patients [8, 13–15]. Moreover, experts recently emphasized the importance of focusing research not only on short-term survival but also on long-term functional outcomes of critically ill stroke patients, in order to improve communication with patients and relatives and to determine the appropriate level of care [8, 16].

The objectives of this study were to describe the clinical features, management, and outcomes of patients admitted to the ICU for acute stroke and to identify predictors of neurological outcome 6 months after ICU admission.

## Methods

This study was approved by the ethics committee of the French Intensive Care Society (CE SRLF 21–09) on January 25, 2021. In accordance with French law on retrospective studies of anonymized healthcare data, informed consent was obtained from all patients and/or their next of kin. This report complies with STROBE guidelines.

### Study design, setting, and population

We retrospectively identified consecutive adults (≥18 years) admitted to the intensive care unit (ICU) of the Nantes University Hospital between January 1, 2014, and December 31, 2018, by searching our hospital database for International Classification of Diseases system (ICD-10) codes indicating stroke (I60.0 to I62.0; I63.0 to I63.9; or I64.0). For patients admitted more than once during the study period, we considered only the first admission. One of us (TC) reviewed the medical chart of each patient thus identified and selected those patients with confirmed stroke. Exclusion criteria were isolated subarachnoid hemorrhage, isolated subdural hematoma, and posttraumatic cerebral hemorrhage.

### Data collection and outcomes

The following data were extracted from the ICU electronic health records (Cerner Millennium, North Kansas City, MI): baseline patient characteristics, including demographics, comorbidities, chronic medications, baseline severity indexes, clinical parameters, admission diagnosis, and stroke characteristics; radiological findings; acute-phase stroke treatments used (thrombolysis or endovascular thrombectomy, neurosurgery, or embolization); occurrence of stroke complications during the ICU stay (hydrocephalus, intracranial hypertension, seizures, status epilepticus, and/or pneumonia); treatment-limitation decisions (withdrawal or withholding of life-supporting interventions) during the ICU stay; and use of mechanical ventilation during the ICU stay. Vital status was recorded at ICU discharge, at hospital discharge, and on days 28 and 180 after ICU admission.

The neurological outcome was assessed based on the modified Rankin Scale (mRS) score on days 28 and 180 after ICU admission, as recorded in the electronic health record. Scores on the mRS can range from 0 to 6, with higher scores indicating greater disability and a score of 6 indicating death. After hospital discharge, mRS scores were assessed by a neurologist during scheduled in-person visits.

### Objectives

The primary objective of the study was to describe the clinical features and outcomes of patients admitted to the ICU for the management of stroke. The secondary objective was to identify predictors of a good 6-month neurological outcome, defined as an mRS score of 0 to 2 (0, no disability; 1, no significant disability; and 2, slight disability) [17, 18].

### Statistical analysis

Continuous variables are described as median [interquartile range] and compared using Wilcoxon’s test. Categorical variables are shown as counts (percent) and compared using Fisher’s exact test. Missing data were ignored. The 6-month neurological outcome was handled as a binary variable (good, i.e., mRS 0–2; or poor, i.e., mRS > 2). Logistic regression analyses were performed to identify variables associated with a good 6-month neurological outcome. The odds ratios (ORs) were computed, with their 95% confidence intervals (95%CIs). A multivariable logistic regression model was then built to identify factors independently associated with a 6-month mRS score of 0–2. The candidate variables were selected based on the literature [16, 19, 20] (age, Glasgow Coma Scale [GCS] score, and hemorrhagic vs. ischemic stroke) and clinical plausibility (persistent pupillary light reflex, body temperature, and acute-phase stroke therapy). All tests were two-sided, with *P* values lower than 5% taken to indicate significant associations. Statistical tests were performed using the R program, version 3.5.0 (R Foundation for Statistical Computing, Vienna, Austria; www.R-project.org/).

## Results

### Study population

**Fig. 1:**
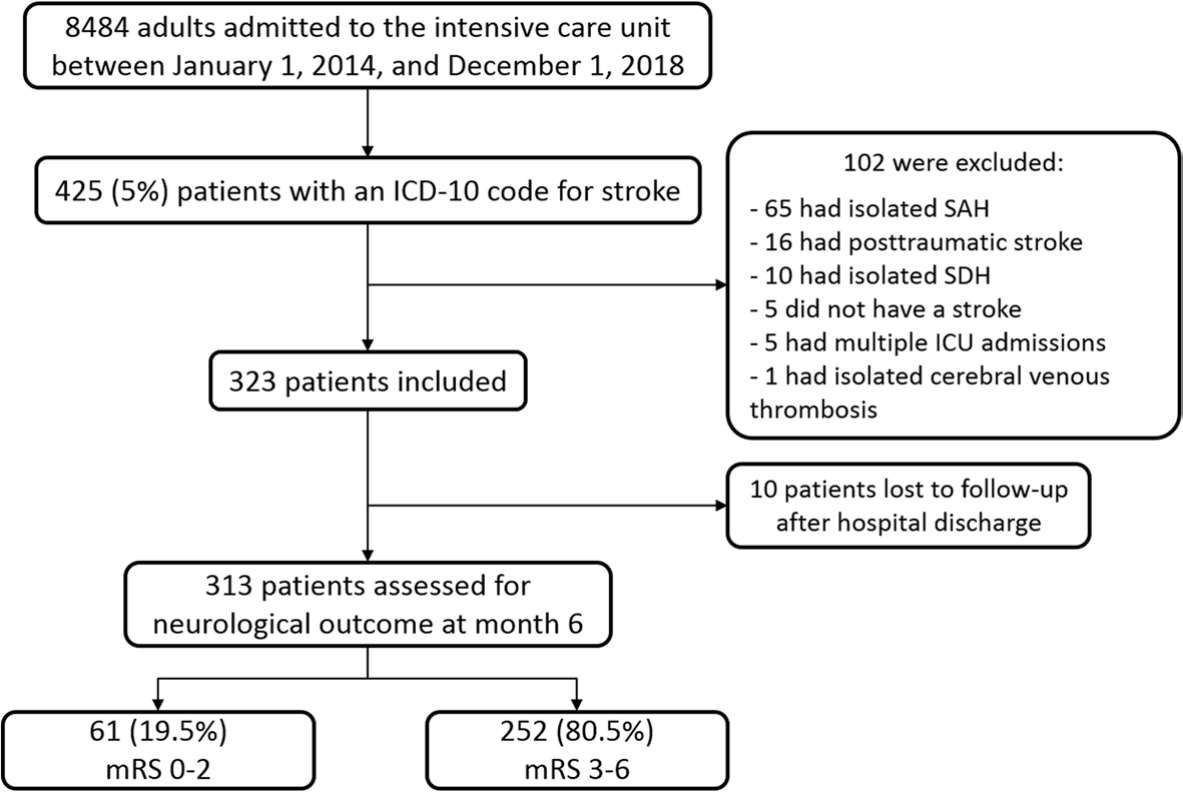
Study flowchart. ICU, intensive care unit; mRS, modified Rankin Scale; SAH, subarachnoid hemorrhage; SDH: subdural hemorrhage

Figure 1 is the flow chart. Table 1 reports the main features of the 323 included patients; among them, 61(18.9%) were considered too sick to benefit from life-sustaining therapies but were admitted to the ICU as potential organ donors.

**Table 1:** Baseline characteristics of the 323 study participants

Variable	All patients (***n*** = 323)
**Demographics**	
Age (years), median [IQR]	67 [54.5–77]
Male, n (%)	173 (53.6)
**Comorbidities, n (%)**	
Hypertension	155 (48)
Coronary artery disease	76 (23.5)
Dyslipidemia	73 (22.6)
Atrial fibrillation	61 (18.9)
Previous stroke	49 (15.2)
Diabetes mellitus	40 (12.4)
**Chronic medications, n (%)**	
Anticoagulation therapy	88 (27.2)
Antiplatelet therapy	65 (20.1)
**Stroke type, n (%)**	
Hemorrhagic	248 (76.8)
Ischemic	75 (23.2)
**Brain site affected, n (%)**	
Ischemic stroke	
Anterior circulation	52 (69.3)
Posterior circulation	23 (30.7)
Cerebral hemorrhage	
Lobar	178 (71.8)
Deep	40 (16.1)
Infratentorial	30 (12.1)
**Clinical variables at ICU admission**	
Glasgow Coma Scale score, median [IQR]	6 [4–10]
Loss of pupillary light reflex, n (%)^a^	112 (34.7)
SAPS II, median [IQR]	54 [35–64]
**Type of ICU admission, n (%)**	
Admission from emergency room or home	283 (87.6)
Transfer from ward	40 (12.4)
**Reason for ICU admission**^**b**^**, n (%)**	
Altered consciousness	281 (87)
GCS 13–15	25 (7.7)
GCS 9–12	39 (12.1)
GCS 3–8	217 (67.2)
Hemodynamic instability	91 (28.2)
Respiratory failure	84 (26)
Cardiac arrest	17 (5.3)

*ICU* intensive care unit, *IQR* interquartile range, *SAPS II* Simplified Acute Physiology Score, version II; *GCS* Glasgow Coma Scale

^a^Missing data for 36 patients

^**b**^Some patients had more than one reason for ICU admission

### ICU management and outcomes

Table 2 reports the treatments and complications. Of the 257 patients who required endotracheal intubation, 252 (98%) were intubated on the first ICU day. Acute-phase stroke therapy was given to 81 (25.1%) patients, including 61 (75.3%) with intracerebral hemorrhage and 20 (24.7%) with ischemic stroke. Seven (2.2%) patients developed acute respiratory distress syndrome (ARDS) and 5 (1.5%) septic shock. Stroke-related complications developed during the ICU stay in 214 (66.3%) patients, the most common being intracranial hypertension and hydrocephalus. In the 124 patients with decisions to limit life-sustaining treatments, the median time from ICU admission to the decision was 3 [1–8] days. Hospital mortality was 87.1% in patients with and 45.2% in patients without treatment-limitation decisions.

**Table 2:** ICU management and outcomes

Variable	All patients (***n*** = 323)
**Acute-phase stroke therapy, n (%)**	81 (25.1)
External ventricular drainage	61 (18.9)
Surgical hematoma drainage	21 (6.5)
Craniectomy	19 (5.9)
Intravenous thrombolysis	9 (2.8)
Endovascular thrombectomy	9 (2.8)
Endovascular coiling of aneurysm	8 (2.5)
**Stroke-related diagnoses, n (%)**	
Intracranial hypertension	188 (58.2)
Acute hydrocephalus	154 (47.7)
Pneumonia	60 (18.6)
Seizures	38 (11.8)
Status epilepticus	10 (3.1)
**Events during the ICU stay**	
Mechanical ventilation, n (%)	257 (79.6)
*Days on mechanical ventilation, median [IQR]*	*3 [2–6]*
Tracheotomy, n (%)	11 (3.4)
Withdrawal or withholding of life-sustaining treatment, n (%)	124 (38.4)
**Length of stay, days, median [IQR]**	
In the ICU	3 [2–7]
In the hospital	5 [2–17]
**Vital status, n (%)**	
Died in the ICU	190 (58.8)
Died in the hospital	198 (61.3)
Died before month 6 (*n* = 313, lost to follow-up n = 10)	202 (64.5)

*ICU* intensive care unit, *IQR* interquartile range

**Fig. 2:**
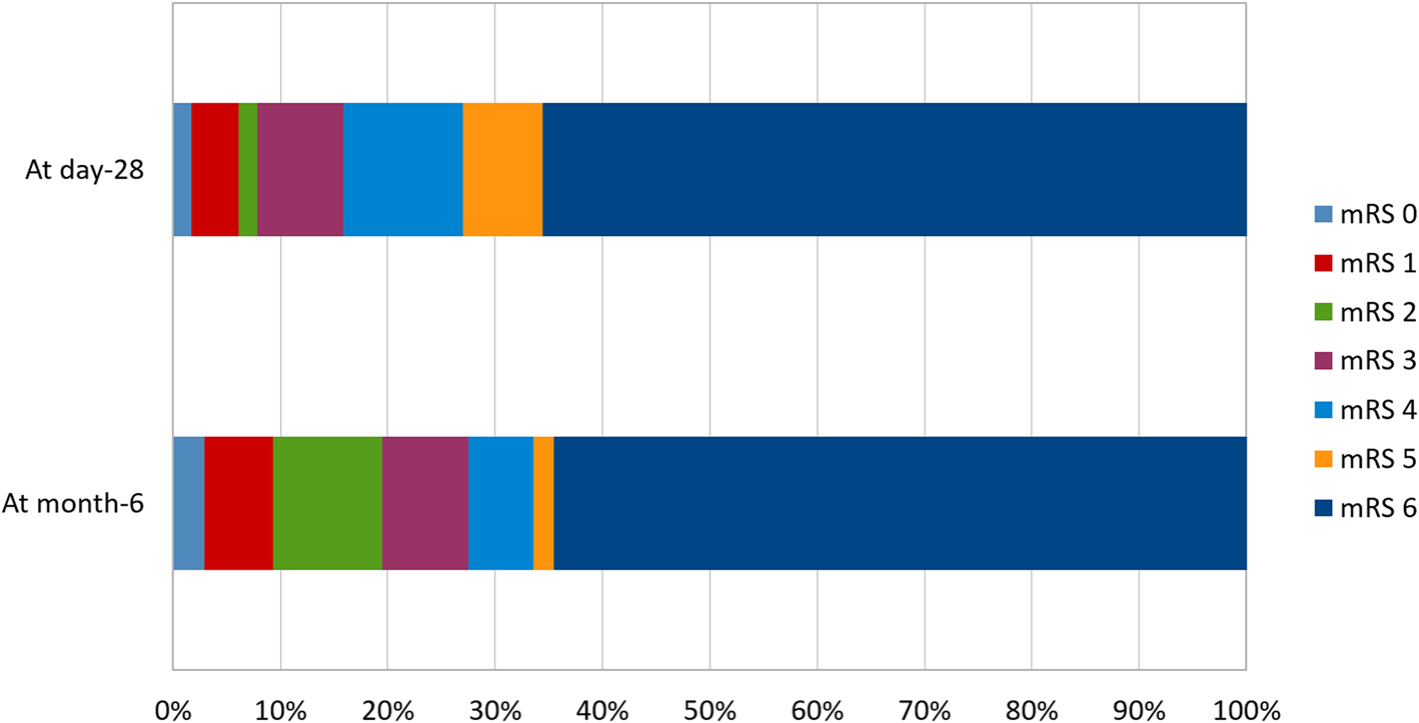
Neurological outcome assessed using the modified Rankin Scale score (mRS) 28 days and 6 months after ICU admission. Missing data: *n* = 26 (8%) on day 28 and *n* = 10 (3.1%) at month 6

Figure 2 shows the mRS scores 28 days and 6 months after ICU admission. On day 28, the mRS was 0–2 in 23 (7.7%) patients, 3–5 in 79 (26.7%) patients, and 6 in 195 (65.6%) patients; the mRS score was missing for 26 patients. At month 6, the mRS was 0–2 in 61 (19.5%) patients, 3–5 in 50 (16.0%) patients, and 6 in 202 (64.5%) patients; the mRS score was missing for 10 patients. The corresponding proportions were 24.1, 19.7, and 56.1%, respectively, in the 252 patients not admitted to the ICU as potential organ donors. Of the 189 patients with no treatment-limitation decisions taken in the ICU, 58 (30.5%), 40 (21%), and 92 (48.5%) had mRS scores of 0–2, 3–5, and 6, respectively, 6 months after ICU admission.

### Factors associated with the 6-month modified Rankin scale score (mRS)

**Fig. 3:**
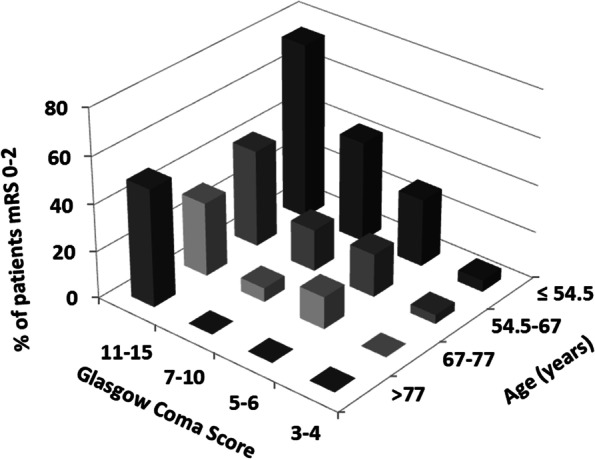
Proportion of patients with a favorable neurological outcome at month 6 according to age and Glasgow Coma Scale score. Favorable neurological outcome was defined as a modified Rankin Scale (mRS) score of 0 (no symptoms), 1 (no significant disability), or 2 (slight disability). mRS, modified Rankin Scale score; SAH: subarachnoid hemorrhage; SDH: subdural hematoma; ICU: intensive care unit

By univariate analysis, variables assessed at ICU admission and associated with a mRS score of 0–2 at 6 months were higher GCS score, persistent pupillary light reflex, higher body temperature, and acute-phase stroke therapy. In contrast, older age, hemorrhagic (versus ischemic) stroke, mechanical ventilation, and greater acute-illness severity (higher SAPSII) were associated with a higher risk of disability or death. Multivariable analysis identified only two independent predictors: older age was negatively associated, and higher GCS score positively associated, with having a mRS between 0 and 2 at 6 months (Table 3 and Fig. 3).

**Table 3:** Logistic regression analyses to identify factors associated with a modified Rankin Scale score (mRS) of 0–2 6 months after ICU admission

Factors	Univariate analysis		Multivariable analysis	
	OR (95%CI)	***P*** value	OR (95%CI)	***P*** value
Age (per year)	0.94 (0.93–0.97)	< 0.01	0.93 (0.89–0.96)	< 0.01
Glasgow Coma Scale score (per point)	1.39 (1.26–1.54)	< 0.01	1.23 (1.07–1.40)	< 0.01
Hemorrhagic (versus ischemic) stroke	0.29 (0.15–0.56)	< 0.01	0.51 (0.17–1.52)	0.22
Persistent pupillary light reflex	12.81 (3.81–43.1)	< 0.01	3.71 (0.82–1.68)	0.09
Body temperature (per additional °C)	1.43 (1.03–1.99)	0.03	1.08 (0.73–1.61)	0.69
Mechanical ventilation	0.09 (0.04–0.18)	< 0.01		
Acute-phase stroke therapy	1.77 (0.99–3.17)	0.05	0.66 (0.22–1.96)	0.45
SAPS II (per point)	0.92 (0.90–0.95)	< 0.01		

*CI* confidence interval, *OR* odds ratio, *SAPS II* Simplified Acute Physiology Score, version II

## Discussion

Two thirds of patients were comatose (GCS score ≤ 8) at ICU admission, nearly 80% required mechanical ventilation while in the ICU, three quarters had hemorrhagic stroke, and only one quarter received acute-phase stroke therapy. Hospital mortality was 61% and less than one-fifth of patients had a good 6-month neurological outcome. The likelihood of survival with a good neurological outcome was lower in older patients and in those with severe consciousness impairment at ICU admission. Neither the type of stroke nor the use of acute-phase stroke therapy was associated with the 6-month neurological outcome.

The epidemiology of stroke requiring ICU admission is unclear. Most studies were conducted in specific stroke subtypes (subarachnoid hemorrhage or acute ischemic stroke) [8, 21], in patients treated with mechanical ventilation [12, 22], or in dedicated stroke units [23]. It has been estimated that 10–20% patients with acute stroke require ICU admission [8–10]. In a German study, mean age of 347 patients admitted to the ICU for acute stroke was 70.8 years, 28.8% of patients were comatose, and 66.6% required intubation [20]. Similarly, in two recent French studies, median age was 63.8–68.2 years, most patients were comatose, and 87 to 100% were intubated [12, 16]. Our findings are consistent with these data.

Over the past two decades, outcomes of stroke have been improved by major therapeutic advances such as reperfusion therapy and decompressive craniectomy [4–6]. In the German study cited above, 38.5% of patients with ischemic stroke underwent reperfusion therapy [20], and in one of the two French studies one-third of patients received reperfusion therapy before ICU admission [16]. In the other French study, conducted over a 10-year period in multiple centers, the proportion of patients given acute-phase stroke therapy increased from 2.9% in 1996–2002 to 21% in 2010–2016 [12]. Our results are in line with these data.

Hospital mortality of critically ill patients with stroke has ranged from 16.3 to 70% [8, 20, 24–30] depending on time period, case mix, ICU admission policies, stroke subtypes, and availability of stroke units. The factors most commonly associated with hospital mortality were older age, use of mechanical ventilation, neurological failure severity at ICU admission, and treatment-limitation policies [8, 13, 14]. Our study reports a high hospital mortality. However, it should be underlined that patients with subarachnoid hemorrhage were excluded from our study and that the majority of our patients had severe neurological failure at admission and were treated with mechanical ventilation. Finally, these figures are similar to those reported by De Montmollin et al. [12]. In one study, treatment-limitation decisions were made three times more often for patients with stroke than for those with other conditions [31]. The proportion in our study was in accordance with earlier reports [32–34], and when we confined our analysis to patients without treatment limitations the proportion with an mRS of 0–2 at 6 months increased only moderately, from 20 to 30%.

In addition to survival, functional outcomes are important to consider [35, 36]. In a British study in 134 patients, only 13.7% had an mRS of 0–2 after 1 year [13]. Of 111 critically-ill patients, less than 30% had an mRS of 0–3 on day 90 [16]. In contrast, of 132 patients admitted for stroke to 16 Spanish ICUs, 43.3% had minimal or no disability at 1 year [14]. However, this study included patients with subarachnoid hemorrhage, and neurological outcomes were good in only 25.0 and 37.1% of patients with ischemic stroke and intracerebral hemorrhage, respectively. In our study, the number of patients mRS 0–2 increased from 23 to 61 between day 28 and month 6, showing some recovery potential with time and underlying the difficulty comparing results at different time points.

Older age and worse consciousness impairment at ICU admission were independently associated with disability and death in our study. Only three earlier studies investigated predictors of functional outcome of ICU stroke patients in the era of reperfusion therapy. In one, a lower GCS score and greater neurological-failure severity independently predicted worse outcomes [16]. In another, the predictors were a lower GCS score, greater acute-illness severity (APACHE II score), and mass effect by computed tomography [13]. Finally, in the remaining study, older age was strongly associated with the neurological outcome after rehabilitation [20].

Our findings indicate that patients admitted to the ICU for stroke are rarely eligible for acute-phase stroke treatments. Data on the effectiveness of these treatments in ICU patients should not be extrapolated from studies in patients who are not critically ill. The independent associations of age and GCS score at ICU admission with the functional outcomes may help physicians inform patients and families and distinguish between patients eligible for continued full-code care and patients for whom transitioning to end-of-life care is more appropriate.

A major strength of our study is the larger sample size compared to earlier similar studies. Moreover, the ICU management of acute stroke remained unchanged during the 5-year recruitment period. Data on the primary outcome were missing for only 3% of patients. The mRS used to assess the primary outcome has been extensively validated and has demonstrated low interobserver and intraobserver variability [37]. Finally, we identified predictors of the 6-month functional outcome. One limitation of our study is the single-center recruitment, which may restrict the general applicability of our findings to similar ICUs in large university hospitals. Selection bias occurred, since we did not include patients with subarachnoid hemorrhage, which has a better outcome compared to hemorrhagic and ischemic stroke [14]. Third, we did not use specific stroke-severity scores (NIHSS [11, 38], intracerebral hemorrhage score [39], or neurosurgical scores [40, 41]), which are not routinely determined in our ICU. Finally, we did not assess quality of life in survivors.

## Conclusion

Most patients admitted to the ICU for stroke had severely impaired consciousness, required mechanical ventilation, and were ineligible for acute-phase stroke therapy. Three fifths of patients died, and only one fifth had a good 6-month neurological outcome. Older age and worse consciousness impairment at ICU admission were independently associated with poorer outcomes. Further studies are needed to identify criteria for choosing the most appropriate level of ICU care in patients with acute stroke.

## Data Availability

The dataset generated during and analyzed during the current study are not publicly available according to the French law but are available from the corresponding author on reasonable request.

## Abbreviations

APACHE II: Acute Physiology And Chronic Health Evaluation II
ARDS: Acute Respiratory Distress Syndrome
CI: confidence interval
GCS: Glasgow Coma Scale
ICD-10: International Classification of Diseases, 10th revision
ICU: intensive care unit
IQR: interquartile range
mRS: modified Rankin Scale
NIHSS: National Institute of Health Stroke Score
OR: odds ratio
SAPSII: Simplified Acute Physiology Score version II
